# Analysis of the Core Genome and Pan-Genome of Autotrophic Acetogenic Bacteria

**DOI:** 10.3389/fmicb.2016.01531

**Published:** 2016-09-28

**Authors:** Jongoh Shin, Yoseb Song, Yujin Jeong, Byung-Kwan Cho

**Affiliations:** ^1^Systems and Synthetic Biology Laboratory, Department of Biological Sciences and KI for the BioCentury, Korea Advanced Institute of Science and TechnologyDaejeon, South Korea; ^2^Intelligent Synthetic Biology CenterDaejeon, South Korea

**Keywords:** acetogens, comparative genomics, conserved pathway, CO_2_ fixation, Wood-Ljungdahl pathway

## Abstract

Acetogens are obligate anaerobic bacteria capable of reducing carbon dioxide (CO_2_) to multicarbon compounds coupled to the oxidation of inorganic substrates, such as hydrogen (H_2_) or carbon monoxide (CO), via the Wood-Ljungdahl pathway. Owing to the metabolic capability of CO_2_ fixation, much attention has been focused on understanding the unique pathways associated with acetogens, particularly their metabolic coupling of CO_2_ fixation to energy conservation. Most known acetogens are phylogenetically and metabolically diverse bacteria present in 23 different bacterial genera. With the increased volume of available genome information, acetogenic bacterial genomes can be analyzed by comparative genome analysis. Even with the genetic diversity that exists among acetogens, the Wood-Ljungdahl pathway, a central metabolic pathway, and cofactor biosynthetic pathways are highly conserved for autotrophic growth. Additionally, comparative genome analysis revealed that most genes in the acetogen-specific core genome were associated with the Wood-Ljungdahl pathway. The conserved enzymes and those predicted as missing can provide insight into biological differences between acetogens and allow for the discovery of promising candidates for industrial applications.

## Introduction

In recent decades, demands for fossil fuel-derived chemicals and energy have rapidly increased, along with concerns about climate change. Currently, ∼80% of world energy is generated via fossil fuel processing, which is responsible for 40% of CO_2_ emissions and global warming (Spigarelli and Kawatra, 2013; Saeidi et al., 2014). Although several methods for replacing fossil fuels have been proposed (Naik et al., 2010), lack of environmental and economic sustainability have demonstrated the technological inability to derive a solution to the climate and energy crisis. As an alternative approach, the gas fermentation process has received attention; it utilizes a unique metabolism in acetogenic bacteria (acetogens), which convert CO_2_ to biofuels (Henstra et al., 2007; Bengelsdorf et al., 2013; Latif et al., 2014).

Acetogens are a physiologically defined group of bacteria that synthesize acetyl-CoA as a central metabolic intermediate from chemolithoautotrophic substrates, such as CO/CO_2_ or H_2_/CO_2_, through acetogenesis (Drake, 1994). Acetogenesis constitutes an appropriate type of microbial metabolism for the substitution of fossil fuels owing to its ability to convert single carbon (C_1_) compounds, such as CO and CO_2_, via the reductive acetyl-CoA pathway to acetyl-CoA, which is referred to as the Wood-Ljungdahl pathway. Owing to this physiological trait, acetogens play key roles in the global carbon cycle (McInerney and Bryant, 1981) by performing the production of large volumes of acetic acid (>10^12^ kg annually; Wood and Ljungdahl, 1991). Moreover, acetogens have been engineered as a novel platform for conversion of waste gasses, such as industrial synthesis gas or syngas, from gasification of biomass into useful multicarbon chemicals (Schiel-Bengelsdorf and Dürre, 2012). This strategy has many advantages over traditional thermochemical processes, such as Fischer-Tropsch synthesis, including operation at lower temperature, lower pressure, higher tolerance of impurities, and flexible syngas-composition utilization (Spigarelli and Kawatra, 2013).

Though acetogens are present in at least 23 different genera (Drake et al., 2006), comprehensive analysis of genes and proteins involved in acetogenesis indicated that acetogens contain conserved physiological properties. The most important shared feature is the conversion of CO_2_ to formate via fixation and to acetyl-CoA, which can be used as a metabolic intermediate for biomass and byproduct synthesis. To elucidate these properties, the biochemistry of the Wood-Ljungdahl pathway and energy conservation systems has been extensively studied (Drake et al., 2008; Ragsdale and Pierce, 2008). In recent years, the enzymatic reactions associated with acetogenesis have been well characterized, especially in *Clostridium autoethanogenum* (Wang et al., 2013; Mock et al., 2015), *Moorella thermoacetica* (Huang et al., 2012; Mock et al., 2014), and *Acetobacterium woodii* (Schuchmann and Müller, 2012; Schuchmann and Muller, 2013; Bertsch et al., 2015).

In addition to the understanding of acetogenesis, elucidation of the molecular mechanisms associated with acetogens has undergone tremendous progress as a result of genome sequencing. The genome sequences of acetogens represent useful information to aid the search for novel enzymes/pathways, generating hypotheses related to energy conservation systems, and accessing evolutionary relationships between species that have not previously been characterized biochemically. For example, studies focusing on construction of *in silico* genome-scale mathematical models, as well as transcriptomics and proteomics investigation of the Wood-Ljungdahl pathway and related energy conservation systems, were undertaken primarily owing to the availability of genome-sequence information (Nagarajan et al., 2013; Islam et al., 2015; Marcellin et al., 2016).

Given the increased volume of genomic information, comparative genomic analysis of acetogens is possible. Among currently available comparative genomic approaches, pan-genome analysis is widely used to construct a framework for estimating genomic diversity of entire repertoires and identifying core genomes (shared by all strains), dispensable genomes (existing in two or more strains), and specific (unique to single strain) gene pools for a species (Tettelin et al., 2005). Conserved and alternative pathways across species provide insight into the biological differences between species (Kelley et al., 2003), allow the discovery of promising target proteins for industrial applications, and create hypotheses regarding missing genes or possible alternatives to current metabolic pathways. Moreover, these findings increase the understanding of genetic differences and related reactions.

In this review, we specifically addressed recent studies on the complete genomes and conserved genes associated with CO/CO_2_ utilization in diverse acetogens. We focused on pathways essential for autotrophic growth, discussed the main features and conservation of metabolic pathways, and addressed the structural differences and relationships between acetogens.

## The Core Genome of Acetogens: Which Genetic Characteristics are Shared Among Acetogens?

Currently, >100 acetogens have been isolated from diverse habitats (Drake et al., 2006). With advances in sequencing technology along with increased biotechnological interest in acetogens, the number of sequenced acetogen genomes has increased every year since the first genome was sequenced. Recently, eight complete genomes (34.7%) were published in 2015, containing five *de novo* sequencing and three resequencing genomes (**Table 1**). In response to the diversely isolated environments and culture conditions, the features of the genomes vary. The length of acetogen genomes range from ∼2.4 to ∼5.7 Mb, with an average length of 3.8 Mb and having GC content between 29.1% and 55.8% (average: 38.5%; **Table 1**). Analysis of sequence annotations revealed that on average, 85.6% of the genomes consist of coding sequences, with approximately 1.1 coding sequence per kb.

**Table 1:** Characteristics of the complete genomes of acetogens.

Strain	Isolation	Temperature	Genome size (bp)	G+C (%)	Number of genes	Number of CDSs	Accession no.	Reference
*Acetobacterium woodii* DSM 1030^∗^	Mud	30°C	4,044,777	39.3	3,649	3,521	CP002987	Poehlein et al., 2012
*Acetohalobium arabaticum* DSM 5501^∗^	Lagoons	37°C	2,469,596	36.6	2,396	2,286	CP002105	Sikorski et al., 2010
*Carboxydothermus hydrogenoformans* Z-2901^∗^	Hotspring	78°C	2,401,520	42	2,495	2,406	CP000141	Wu et al., 2005
*Clostridium aceticum* DSM 1496^∗^	Mud	30°C	4,201,318	35.3	3,847	3,705	CP009687	Poehlein et al., 2015b
*Clostridium autoethanogenum* DSM 10061^∗^	Rabbit faeces	37°C	4,352,205	31.1	3,983	3,741	CP006763	Brown et al., 2014
*Clostridium autoethanogenum* DSM 10061	Rabbit faeces	37°C	4,352,446	31.1	4,069	3,964	CP012395	Humphreys et al., 2015
*Clostridium carboxidivorans* P7^∗^	Lagoons	37°C	5,732,880	29.9	5,167	5,004	CP011803	Li et al., 2015
*Clostridium ljungdahlii* DSM 13528^∗^	Chicken yard waste	37°C	4,630,065	31.1	4,234	4,081	CP001666	Köpke et al., 2010
*Clostridium scatologenes* ATCC 25775^∗^	Soil	37°C	5,749,410	29.6	5,183	4,974	CP009933	Zhu et al., 2015
*Clostridium sticklandii* DSM 519	Mud	37C	2,715,461	33.3	2,625	2,476	FP565809	Fonknechten et al., 2010
*Eubacterium limosum* KIST 612^∗^	Anaerobic digester fluid	37°C	4,316,707	47.5	4,089	3,966	CP002273	Roh et al., 2011
*Eubacterium limosum* SA11	Sheep lumen	37°C	4,150,332	47.4	3,902	3,805	CP011914	Kelly et al., 2016
*Moorella thermoacetica* ATCC 39073^∗^	Horse faeces	55°C	2,628,784	55.8	2,613	2,463	CP000232	Pierce et al., 2008
*Moorella thermoacetica* DSM 521	Horse faeces	55°C	2,527,564	55.9	2,518	2,405	CP012369	Poehlein et al., 2015a
*Moorella thermoacetica* DSM 2955	Horse faeces	55°C	2,623,349	55.8	2,609	2,508	CP012370	Bengelsdorf et al., 2015
*Clostridium difficile* 630^∗^	Human intestine	37°C	4,290,252	29.1	3,971	3,756	AM180355	Sebaihia et al., 2006
*Clostridium difficile* CD196	Human intestine	37°C	4,110,554	28.7	3,526	3,487	FN538970	Stabler et al., 2009
*Clostridium difficile* M120	Human intestine	37°C	4,047,729	28.7	3,707	3,502	FN665653	He et al., 2010
*Clostridium difficile* 630	Human intestine	37°C	4,274,806	29	3,972	3,794	CP010905	Riedel et al., 2015
*Clostridium difficile* 630 Deltaerm	Human intestine	37°C	4,293,049	29.1	3,990	3,816	LN614756	van Eijk et al., 2015
*Thermacetogenium phaeum* DSM 12270^∗^	sludge	60°C	2,939,057	53.9	2,894	2,766	CP003732	Oehler et al., 2012
*Thermoanaerobacter kivui* LKT-1^∗^	Lake sediment	65°C	2,397,824	35	2,425	2,198	CP009170	Hess et al., 2014
*Treponema primitia* ZAS-2^∗^	Termite	30°C	4,059,867	50.8	3,536	3,427	CP001843	Rosenthal et al., 2011

*Genome sequences analyzed in this paper are indicated in asterisk (^∗^).*

Based on these complete acetogen genomes, comprehensive genome analysis is possible to understand the functionality and specificity conserved among autotrophic acetogenic bacteria (Hayashi et al., 2001; Ohnishi et al., 2001). For this purpose, we selected 14 strains that have been experimentally confirmed as capable of converting acetyl-CoA from CO/CO_2_ and, thus, from inorganic carbon through the Wood-Ljungdahl pathway (**Table 1**). Although *Carboxydothermus hydrogenoformans* and *Thermacetogenium phaeum* are carboxydotrophic hydrogenogenic and syntrophic acetate-oxidizing bacteria, respectively, unlike model acetogens, their acetogenic growth has been reported (Hattori et al., 2000, 2005; Henstra and Stams, 2011; Haddad et al., 2013). On the other hand, the capability of *Clostridium sticklandii* DSM 519 for autotrophic growth on C_1_ substrates via the Wood-Ljungdahl pathway was not confirmed (Fonknechten et al., 2010); therefore, this strain was excluded in this analysis.

**FIGURE 1:**
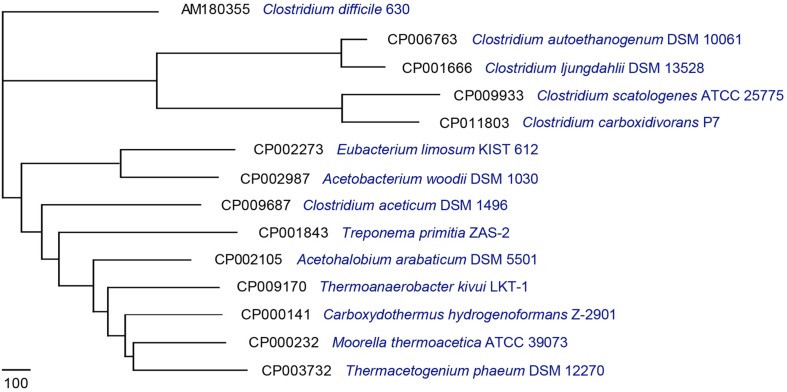
**Pan-genome tree consisting of 14 acetogens.** A pan-genome tree consisting of 14 acetogens was constructed using the neighbor-joining method core-genome-determined values.

For downstream analysis, 14 complete acetogen genome sequences were obtained from the National Center for Biotechnology Information database^1^ (**Table 1**). Pan-Genomes Analysis Pipeline (PGAP-1.12; Zhao et al., 2012) identified functional genes presented in all strains (core genome), two or more strains (dispensable genomes), and unique strains (specific genomes; Tettelin et al., 2005). For comparative analysis, the MultiParanoid method was used to analyze cluster orthologs and inparalogs shared by multiple genomes based on sequence similarity (Alexeyenko et al., 2006; Zhao et al., 2012). Additionally, BLASTP was used to determine similarities between protein sequences and filter results by setting minimum scores at 50 and *E*-values to 10^-10^. The obtained result was clustered using the Markov cluster algorithm (Enright et al., 2002). To understand the evolutionary relationships among these acetogens, a pan-genome tree was constructed (**Figure 1**) based on the pan-genome dataset and neighbor-joining method (Zhao et al., 2012). All sister groups were clustered by the same genera or optimal temperature conditions. In contrast to the 16S-based phylogenetic tree (Bengelsdorf et al., 2013), the strain exhibiting the least amount of evolutionary change from a common ancestor was *Clostridium difficile*. *M. thermoacetica* (strain AMP) was previously reported to show atypical hydrogenogenic metabolism (Jiang et al., 2009), and the pan-genome tree also showed evolutionary closeness among *Ca. hydrogenoformans, T. phaeum*, and *M. thermoacetica* (**Figure 1**). These results suggested that functional gene composition of *M. thermoacetica* is similar to *Ca. hydrogenoformans.*

**FIGURE 2:**
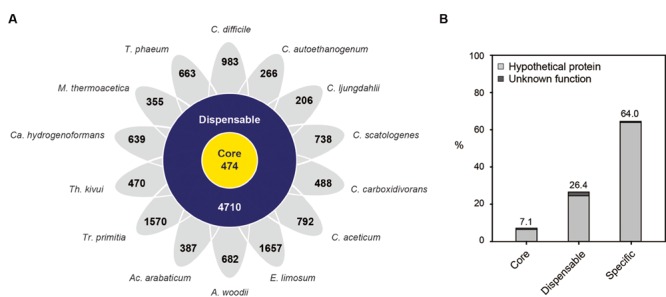
**Pan-genome analysis of acetogens. (A)** The number of core, dispensable, and specific genomes of each strain. Abbreviations: A, *Acetobacterium*; Ac, *Acetohalobium*; Ca, *Carboxydothermus*; C, *Clostridium*; E, *Eubacterium*; M, *Moorella*; T, *Thermoacetogenium*; Tr, *Treponema*; Th, *Thermoanaerobacter*. **(B)** Proportion of hypothetical and uncharacterized proteins in the groups of core, dispensable, and specific genes was calculated and displayed as follows: hypothetical proteins, light gray; unknown proteins, dark gray.

According to comparative genome analysis, a total of 15,079 orthologous groups with 50,178 genes were identified, consisting of 474 core gene groups with 12,457 genes, 4710 dispensable gene groups with 27,825 genes, and 9896 specific genes identified (**Figure 2A**; Supplementary Table S6). Core genes were well annotated, with 92.9% of genes. However, the number of specific genes in each organism varied from 206 to 1657, with 64.0% of the specific genes identified as having hypothetical functions (**Figure 2B**). Additionally, the number of specific genes did not correlate with the size of the genome, which is in contrast to the correlation between the number of genes and the size of the genome. For example, the genome of *Clostridium ljungdahlii* is the third largest (4.6 Mb), but its number of specific genes is 206, which is the least number of genes in the set. Additionally, 266 specific genes, which was the second least number of genes in the set, were found in *C. autoethanogenum*, having the fourth largest (4.3 Mb) genome.

To decipher the 474 core genes of the 14 acetogenic bacteria, functionally grouped networks of enriched categories were generated for the biological interpretation of core genes using ClueGo version 2.2.4 (Saito et al., 2012), which is a widely used Cytoscape version 3.3.0 (Shannon et al., 2003) plugin. For this analysis, *C. autoethanogenum* data was used as the standard, because *C. autoethanogenum* was recently confirmed systematically by transcriptome and proteome analysis of the Wood-Ljungdahl pathway (Marcellin et al., 2016). Gene Ontology (GO) terms (GO:0030634; Biological Process, carbon fixation by acetyl-CoA pathway) and Kyoto Encyclopedia of Genes and Genomes (KEGG) pathways (M00377; Pathway module, Wood-Ljungdahl pathway) were manually added along with the published experimental evidence (Marcellin et al., 2016) (Supplementary Table S1).

**FIGURE 3:**
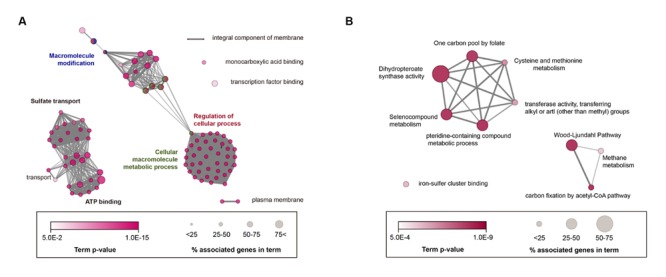
**Enrichment map of GO (Gene Ontology) terms and KEGG (Kyoto Encyclopedia of Genes and Genomes) pathways in the core acetogen genome. (A)** Annotation-term network of core acetogen genomes. **(B)** Acetogen-specific core genomes using functional enrichment analysis. KEGG and GO terms, including biological process, molecular function, and cellular component, were represented together as nodes, and node sizes represent the genes percentage association with each term. Significantly related terms were highly contacted, and functionally related nodes were partially overlapped. The most significant terms were only annotated in groups. A Bonferroni corrected *p* < 0.05 was considered the cut-off criterion. Term enrichment significance was represented by color.

As a result, 95 GO terms were significantly enriched and categorized into 10 groups according to their kappa scores (**Figure 3A**). Overall, highly connected groups were assigned to adenosine triphosphate (ATP) binding, macromolecule modification and sulfate transport, cellular macromolecule metabolic process, and regulation of cellular process as group-leading terms (**Figure 3A**). Additionally, five sub-groups were involved in membrane component, monocarboxylic acid binding, transcription-factor binding, and transport and plasma membrane (**Figure 3A**; Supplementary Table S2). Therefore, GO analysis showed that the core genome was significantly correlated with a number of essential cellular functions, similar to most bacteria (Gil et al., 2004). To examine the acetogenic characteristics, core genome was trimmed by non-acetogenic core genome, which contains five non-acetogens phylogenetically close to 14 selected acetogenic bacteria (Supplementary Figure S1). Based on enrichment *p*-values, 27 GO terms and 8 KEGG pathways were enriched (Supplementary Table S3) and functionally categorized into 12 groups (Supplementary Figure S2). The most linked functional groups were assigned to cysteine and methionine metabolism, monobactam biosynthesis, small molecule biosynthetic process, Mo-molybdopterin cofactor biosynthetic process, iron chelate transport, and the Wood-Ljungdahl pathway. This result is in agreement with related acetogenesis and cofactor biosynthetic pathways involved in the Wood-Ljungdahl pathway.

To further investigate unique core genes found in acetogens, the core genome was filtered using genomes of non-acetogenic anaerobic bacteria. In this analysis, the complete genome of *Clostridium butyricum* KNU-L09 was used, which is a strictly anaerobic, non-acetogenic bacteria that is phylogenetically similar to *C. difficile* 630 (Supplementary Figure S1). According to the functional annotation network of the acetogen-specific core genome, five KEGG pathways and five GO terms were specifically enriched (**Figure 3B**; Supplementary Table S4). Acetogen-specific functional networks consisted of 13 genes annotated as methionine synthase, CO dehydrogenase/acetyl-CoA synthase (CODH/ACS), ferredoxins, and a subunit of formylmethanofuran dehydrogenase. Thus, acetogen-specific functional networks were involved in specific molecular functions, such as iron-sulfur cluster-binding transferase activity and dihydropteroate-synthase activity, and biological processes, such as carbon fixation by the acetyl-CoA pathway and the pteridine-containing compound metabolic process. Interestingly, 12 of the 13 genes (92.3%) were highly associated with the Wood-Ljungdahl pathway. Of the 12 genes, six were located in a single gene cluster encoding the Wood-Ljungdahl pathway (CAETHG_1606-CAETHG_1621), while the other six genes were additional copies of those genes. Another gene specifically conserved in acetogens was the tungsten-containing formylmethanofuran dehydrogenase subunit E (*fwdE*), which catalyzes the first reduction of CO_2_ in methanogens (Hochheimer et al., 1998). However, the other genes encoding tungsten formylmethanofuran dehydrogenase (*fwdABCD*), which often form an operon with *fwdE*, were absent in all 14 acetogen genomes. This protein encoded by *fwdE* contains a zinc-β-ribbon domain, suggesting that it plays a role in transcriptional regulation as a DNA-binding protein; however, its exact role in acetogenesis remains unclear.

## Biosynthesis of Acetate From Co/Co_2_: The Wood-Ljungdahl Pathway

Based upon the analysis of the acetogen-specific core genome, the genes related to the Wood-Ljungdahl pathway were highly conserved as hallmarks of acetogens. This pathway involves the reduction of two CO_2_ molecules into one acetyl-CoA with several coenzymes and electron carriers (Drake and Daniel, 2004; Ragsdale, 2008), and it is highly interconnected with energy conservation systems to overcome the same thermodynamically unfavorable reaction. Nevertheless, the pathway is the most efficient of the all CO_2_-fixation pathways, including the Calvin cycle, the reductive tricarboxylic acid cycle, and the hydroxypropionate cycle (Fast and Papoutsakis, 2012). Moreover, the arrangement of genes related to the Wood-Ljungdahl pathway was well conserved with phylogenetic correlation in their genomes (Poehlein et al., 2015c). In this review, the Wood-Ljungdahl pathway was functionally separated into three core groups. The first core group encodes enzymes responsible for reducing CO_2_ to formate. The second core group consists of the methyl- and the carbonyl-branch enzymes. The last core group is composed of acetate-producing genes.

## The Wood-Ljungdahl Pathway Core Group I: Co_2_ to Formate

**FIGURE 4:**
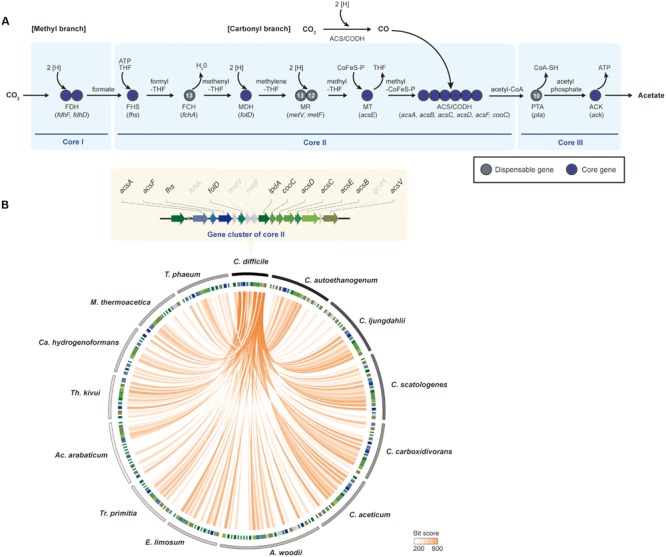
**The Wood-Ljungdahl pathway. (A)** The methyl- and the carbonyl-branches in the Wood-Ljungdahl pathway. The Wood-Ljungdahl pathway is shown with genes that are represented as core genes (blue circles) and dispensable genes (dark gray circles). The numbers within the circles represent the number of strains that have corresponding genes in other strains. Abbreviations: THF, tetrahydrofolate; CoFeS-P, corrinoid [Fe-S] protein; FDH, formate dehydrogenase; FHS, formyl-tetrahydrofolate synthase; FCH, formyl-cyclohydrolase; MDH, methylene-THF dehydrogenase; MR, methylene-THF reductase; MT, methyltransferase; ACS/CODH, carbon monoxide dehydrogenase/acetyl-CoA synthase; PTA, phosphotransacetylase; ACK, acetate kinase. **(B)** Comparison of the Wood-Ljungdahl pathway genes between *Clostridium difficile* 630 and 13 other acetogenic bacteria used in pan-genome analysis. Track 1 (the outermost) represents boundaries of each bacterium. The clockwise order of the genera is based on the phylogenetic tree in **Figure 1**. Track 2 represents the Wood-Ljungdahl pathway genes, the colors of which are indicated in the upper panel. Orange lines link the genes that have *e*-values <10^-6^. Abbreviations: A, *Acetobacterium*; Ac, *Acetohalobium*; Ca, *Carboxydothermus*; C, *Clostridium*; E, *Eubacterium*; M, *Moorella*; T, *Thermoacetogenium*; Tr, *Treponema*; Th, *Thermoanaerobacter*.

The first reaction of acetogenesis is the reduction of CO_2_ to formate by two-electron reduction, which is catalyzed by selenocysteine- or non-selenocysteine-containing formate dehydrogenase (FDH) in a ferredoxin- or NADH-dependent reaction (Ljungdahl and Andreesen, 1978; Gollin et al., 1998; Schuchmann and Muller, 2013; Wang et al., 2013). Genes associated with the reaction are well conserved in all acetogens. According to genome-comparison analysis, two genes encoding selenocysteine-containing FDH (*fdhF*) and FDH-accessory protein (*fdhD*) are well conserved in core group I (**Figure 4A**). Despite conservation of *fdhF* and *fdhD*, a number of *fdh* gene copies are different in all of the genomes. For instance, *fdhF* and *fdhD* were located as a single gene cluster in the *C. difficile* genome. However, three copies of *fdhF* were found in *C. ljungdahlii* and *C. autoethanogenum*. Similar to the genes encoding seleno-containing FDH, the genes encoding non-selenocysteine residues containing FDH are also well conserved in the acetogen genomes. Although the selenoproteins are mutant forms of FDH that differ only in the presence of selenium instead of sulfur at the active site, seleno-containing FDHs exhibit higher catalytic rates relative to non-selenocysteine FDHs (Stadtman, 1991; Matson et al., 2010). However, non-selenocysteine FDH may be useful for acetogenesis in selenium-free environments.

Although the *fdh* genes are highly conserved, electron-delivery systems involved in this reaction differ, owing to the diversity of electron acceptors associated with FDH (Schuchmann and Müller, 2014). For example, *A. woodii* and *Clostridium aceticum* have four or three hydrogenase modules, respectively, which are located in a gene cluster with the selenocysteine-containing *fdh* genes (Poehlein et al., 2012, 2015c; Schuchmann and Muller, 2013). In this process, *A. woodii* uses H_2_ as an electron donor for CO_2_ reduction, referred to as hydrogen-dependent CO_2_ reductase, which can be energetically more advantageous as compared with utilizing energy intermediates by not expending a substrate for the chemiosmotic gradient (Schuchmann and Muller, 2013). *C. autoethanogenum* and *C. ljungdahlii* also have complexes of ferredoxin and NAD-dependent [FeFe]-hydrogenases for CO_2_ reduction, which are located near an *fdh* gene cluster encoding selenocysteine-containing FDH (Nagarajan et al., 2013; Wang et al., 2013).

## The Wood-Ljungdahl Pathway Core Group Ii: Formation of Acetyl-CoA

Formate is subsequently converted to acetyl-CoA by a series of reactions catalyzed by the enzymes of the methyl branch of the Wood-Ljungdahl pathway. Core group II was composed of all key enzymes in the methyl and carbonyl branches (**Figure 4A**). In the methyl branch, formyl-tetrahydrofolate (THF) synthase (FHS) converts formate to formyl-THF by investing one molecule of ATP. For the next two steps, formyl-THF cyclohydrolase (FCH) and methylene-THF dehydrogenase (MDH) consecutively catalyze the converted THF into methenyl-THF, then to methylene-THF, which is then converted to methyl-THF and methyl-CoFeSP by using methylene-THF reductase (MR, two subunits of methylene-THF reductase; *metV* and *metF*) and methyltransferase (MT, two subunits of corrinoid/Fe-S protein; *acsC* and *acsD*, methyltransferase: *acsE*), respectively. For the carbonyl branch, CO_2_ becomes CO via catalysis by the CODH/ACS complex (CODH: *acsA, acsF*, and *cooC*; ACS: *acsB*). Using the same enzyme, the two molecules, methyl-CoFeSP and CO, combine into acetyl-CoA.

Nine genes encoding FHS, MDH, MT, CODH, and ACS were well conserved in all 14 acetogens. However, two genes that encode FCH and two MR subunits were determined to be dispensable genes. One of the four dispensable genes, *fchA*, is responsible for converting formyl-THF into methyl-THF. In order to perform a similarity search of *fchA* throughout the other genomes, the *fchA* sequence from *C. difficile* was used, and it was determined that *fchA* from 13 acetogen genomes was highly conserved, although the enzyme was only absent in the *M. thermoacetica* genome (Pierce et al., 2008). According to a previous study, in *M. thermoacetica*, the cyclization of formyl-THF and the reduction of methenyl-THF were observed being catalyzed by MDH by substituting FCH (O’Brien et al., 1973; Pierce et al., 2008), which is not a core gene in the Wood-Ljungdahl pathway. Although the *fchA* gene is not a core gene set, the biochemical reaction associated with conversion of formyl-THF to methylene-THF is a conserved step in all acetogens for acetogenesis.

Other dispensable genes included *metF* and *metV* that encode MR. These redox enzymes contain iron-sulfur clusters and utilize reduced forms of electron carriers (ferredoxin or NADH) as electron donors. They reduce methylene-THF to methyl-THF using different enzyme complexes (Clark and Ljungdahl, 1984; Park et al., 1991). In this step, enzymatic diversity denoted by related-subunit compositions was reported among acetogens (Mock et al., 2014; Bertsch et al., 2015; Jeong et al., 2015). In *A. woodii*, a trimeric enzyme-complex system was detected for methyl-THF conversion, consisting of *metF, metV*, and *rnfC2* (Bertsch et al., 2015). In the gene cluster, RnfC2 accepts an electron from the reduced form of NADH and then transfers the electron to reduce methylene-THF. However, the MR gene cluster consists of a heterohexameric complex with electron-bifurcating heterodisulfide reductase (*hdrA, hdrB*, and h*drC*), *metV*, and *mvhD* in *M. thermoacetica* (Mock et al., 2014). Additionally, the heterohexameric complex does not catalyze NADH-dependent methylene-THF reduction, but utilizes some form of second-electron acceptor. Although genes of redox enzymes were highly conserved, a configuration of actual enzymatic reactions will be quite different. According to the results of the comparative analysis, only *metV* is absent in *Acetohalobium arabaticum*, and both genes encoding MR are missing in *Treponema primitia*. In other bacteria, *Thermus thermophilus* HB8 and *Escherichia coli* K12 utilize only *metF* to catalyze the methylene tetrahydrofolate reductase reaction (Guenther et al., 1999; Igari et al., 2011). Perhaps the conversion of 5,10-methylenetetrahydrofolate to 5-methyltetrahydrofolate in *Ac. arabaticum* may function as an MR reaction in *Escherichia coli* and *T. thermophiles* containing only *metF*. The *Ac. arabaticum metF* gene consists of methylenetetrahydrofolate reductase and methylene-tetrahydrofolate reductase C-terminal domains and is 663 base pairs longer than the *A. woodii metF* gene. Given the presence of the *metV* domains, the *metF* gene in *Ac. arabaticum* is capable of solely catalyzing MR reactions to reduce methylene-THF. However, alternative pathways for the missing subunits involved in the MR reaction in *Tr. primitia* remain unknown.

The last dispensable gene in core group II is *gcvH*, encoding glycine-cleavage system H protein in the glycine cleavage/synthesis pathway, whose functional role in the Wood-Ljungdahl pathway remains unclear. The glycine cleavage/synthesis pathway consists of four proteins; however, only *gcvH* and *lpdA*, which encodes dihydrolipoamide dehydrogenase, are acetogens. All of the genes encoding this pathway are found in *C. sticklandii* (Fonknechten et al., 2010). Although the genes encoding the complete Wood-Ljugdahl pathway are present in the genome, *C. sticklandii* is unable to utilize CO_2_ as a substrate. One proposed hypothesis is that due to the presence of all glycine cleavage/synthesis complexes, an efficient electron acceptor substitutes for the role of CO_2_, which leads to shutdown of the methyl-branch of the Wood-Ljungdahl pathway (Fonknechten et al., 2010). Although *lpdA* is conserved in 14 acetogens, *gcvH* is absent in core group II due to the risk of shutting down the Wood-Ljungdahl pathway.

Aside from enzymatic diversity, conserved genes from core group II showed a tendency to co-localize in the genomes (**Figure 4B**). Although acetogens are phylogenetically diverse, conserved genes encoding FHS or CODH/ACS complexes are co-localized in acetogen genomes (Bruant et al., 2010; Poehlein et al., 2015c). In the least evolutionarily changed *C. difficile* genome (**Figure 1**), the Wood-Ljungdahl pathway enzymes are located in one gene cluster (**Figure 4B**), which has been reported (Bruant et al., 2010; Köpke et al., 2013). Although two copies of *lpdA* were found, only one copy of each core gene was detected. In all *Clostridium* genera of acetogenic bacteria, the Wood-Ljungdahl pathway gene cluster with the same order of genes was conserved (**Figure 4B**). Beside the *Clostridium* genera, the methyl- and carbonyl-branch-encoding genes presented as multiple copies. *A. woodii* and *Eubacterium limosum* are phylogenetically related and contain two gene clusters encoding the Wood-Ljungdahl pathway, which is composed of both the methyl and the carbonyl branches. Additionally, duplication of *acsE* explains the rapid growth rate under autotrophic conditions in both strains (Blach et al., 1977; Tschech and Pfennig, 1984; Sharak Genthner and Bryant, 1987). Interestingly, throughout all 14 acetogens, *acsB, acsC, acsD, acsE*, and *acsF* genes were always located as a gene cluster (**Figure 4B**). Thus, the highly conserved CODH/ACS complex indicated that the complex functions most efficiently when the genes form a gene cluster. Under such circumstances, gene clusters reflect evolutionary changes in pathways and associated taxonomy, while the phylogenetic tree describes the evolution of acetogenic bacteria.

## The Wood-Ljungdahl Pathway Core Group Iii: Acetyl-CoA to Acetate

All acetogens have an ability to produce acetate via acetogenesis as a core feature (Drake et al., 2008). In many acetogenic bacteria, phosphotransacetylase (*pta*) and acetate kinase (*ack*) genes were found as a single operon, similar to that observed in *C. ljungdahlii*, and *C. autoethanogenum* (Köpke et al., 2010; Brown et al., 2014). In the 14 acetogen genomes, the *ack* gene was categorized as a core gene, but the *pta* gene was classified as a dispensable gene. The acetate-production operon, which consisted of the *pta* and *ack* genes, was found in *C. autoethanogenum, C. ljungdahlii, Clostridium scatologenes, Clostridium carboxidivorans, Thermoanaerobacter kivui, Ca. hydrogenoformans*, and *T. phaeum*. However, in *A. woodii* and *Tr. primitia*, the *ack* and *pta* genes were scattered in the genomes and not located as a gene cluster. Additionally, the *pta* gene was unidentified in four acetogen genomes: *C. difficile, C. aceticum, E. limosum*, and *M. thermoacetica*. It was suggested that an alternative protein for *pta* is phosphotransbutyrylase (*ptb*; Köpke et al., 2013; Poehlein et al., 2015b) and butyrate kinase (*buk*), which are located on a single operon and can bind to both acetyl-CoA and butyryl-CoA, or propanediol utilization protein (*pduL*), which exhibits transacetylase function (Pierce et al., 2008; Köpke et al., 2010; Poehlein et al., 2015b). In contrast to *pta*, the *ack* gene was found as a single copy and exhibited high similarity in all strains, except *Ac. arabaticum*, which has two *ack* genes.

## Central Intermediates of Autotrophic Growth: Acetyl-CoA and Pyruvate

As an essential cellular function in all bacteria, biomass and byproducts must be derived from acetyl-CoA. For bacterial growth under autotrophic conditions, the central precursor can only be synthesized from C_1_ compounds via the Wood-Ljungdahl pathway, which plays an important role in cell proliferation. According to a previous study, the proportion of carbon flux toward biomass was predicted as 5% of total carbon flux during autotrophic fermentation (Fast and Papoutsakis, 2012).

**FIGURE 5:**
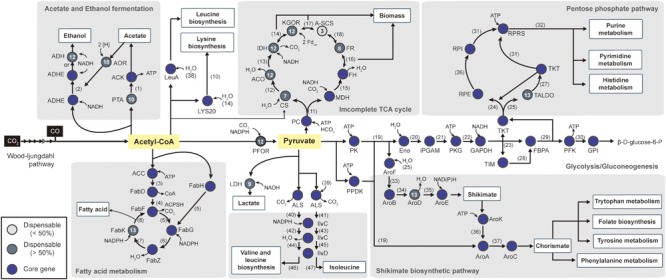
**Pathway map of central carbon metabolism.** Starting from Acetyl-CoA, the pathway includes 52 biochemical steps catalyzed by enzymes (see Supplementary Table S5 to see the complete enzyme name). The total pathway is shown with genes that are represented as core genes (blue circles), lesser conserved dispensable genes (<50%, light gray circles), and highly conserved dispensable genes (>50%, dark gray circles). The numbers within the circles represent the number of strains that have corresponding genes in other strains. The following metabolites are represented by number: (1) Acetyl phosphate, (2) Acetaldehyde, (3) Malonyl-CoA, (4) Malonyl-[acyl-carrier protein], (5) Acetoacetyl-[acyl-carrier protein], (6) (R)-3-Hydroxybutanoyl-[acyl-carrier protein], (7) But-2-enoyl-[acyl-carrier protein], (8) Butanoyl-[acp], (9) Acetyl-[acyl-carrier protein], (10) Homocitrate, (11) Oxaloacetate, (12) Citrate, (13) Isocitrate and aconitate, (14) 2-Oxoglutarate, (15) Malate, (16) Fumarate, (17) Succinyl-CoA, (18) Succinate, (19) Phosphoenol-pyruvate, (20) 2-Phospho-D-glycerate (21) 3-Phospho-D-glycerate, (22) 1,3-Bisphospho-D-glycerate, (23) D-Glyceraldehyde 3-phosphate, (24) D-Xylulose-5P, (25) D-Erythrose-4P, (26) D-Ribulose 5-phosphate, (27) D-Sedoheptulose 7-phosphate (28) dihydroxyacetone phosphate (DHAP), (29) D-Fructose-1,6-bis, (30) D-Fructofuranose 6-phosphate, (31) D-Ribose-5P, (32) 5-Phospho-alpha-D-ribose 1-diphosphate, (33) 3-Deoxy-D-arabino-hept-2-ulosonate 7-phosphate, (34) 3-Dehydroquinate, (35) 3-Dehydroshikimate, (36) Shikimate 3-phosphate, (37) 5-Enolpyruvyl-shikimate 3-phosphate, (38) 3-Methyl-2-oxobutanoate, (39) 2-Oxoburanoate, (40) (S)-2-Acetolactate, (41) (S)-2-Aceto-2-hydroxybutanoate, (42) 3-Hydroxy-3-methyl-2-oxobutanoic acid, (43) (R)-3-Hydroxy-3-methyl-2-oxopentanoate, (44) (R)-2,3-Dihydroxy-3-methylbutanoate, (45) (R)-2,3-Dihydroxy-3-methylpentanoate, (46) 3-Methyl-2-oxobutanoic acid, (47) (S)-3-Methyl-2-oxopentanoic acid.

Acetate and ethanol are common products generated by acetogenic fermentation, and the production of acetate coupled to ATP synthesis is associated with the Wood-Ljungdahl pathway. Following acetate production, acetate is reduced to acetaldehyde via an aldehyde:ferredoxin oxidoreductase reaction with reduced ferredoxin, and the corresponding gene is categorized as a dispensable gene. Acetyl-CoA can also be converted to acetaldehyde by bifunctional aldehyde/alcohol dehydrogenase (Leang et al., 2013), which was conserved in all 14 acetogens. Additional reduction of acetaldehyde can generate ethanol by the same aldehyde/alcohol dehydrogenase or alcohol dehydrogenase (**Figure 5**; Supplementary Table S5). Although the alcohol dehydrogenase or aldehyde/alcohol dehydrogenase enzymes responsible for ethanol production are encoded in their genomes, ethanol production was reported in only four strains under autotrophic conditions. Three strains, *C. autoethanogenum* (Köpke et al., 2011), *C. ljungdahlii* (Köpke et al., 2010), and *C. carboxidivorans* (Liou et al., 2005; Bruant et al., 2010), are capable of producing ethanol as the main product, and *C. scatologenes* (Liou et al., 2005) is able to produce ethanol at low levels. Although genetic mechanisms for ethanol production are present, ethanol production by other strains was not reported under autotrophic conditions. Possible explanations are that these strains lack functional efficiency of the aldehyde:ferredoxin oxidoreductase reaction (putative formaldehyde:Fd oxidoreductase) or presence of bioenergetic constraints (Bertsch and Müller, 2015; Mock et al., 2015).

In addition to alcohol production, acetyl-CoA can be used for fatty acid, leucine, and lysine biosynthesis in one of the most conserved pathways in bacteria. Acetyl-CoA can be utilized directly for fatty acid biosynthesis by seven conserved genes. Although six of the genes were classified as core genes, enoyl-acyl carrier-protein reductase (*fabK*, EC 1.3.1.9) was identified as being dispensable due to its being absent in *Tr. primitia* (**Figure 5**).

To biosynthesize nucleic acids, amino acids, and essential cofactors, three-carbon pyruvate was used as a central metabolite in several pathways for autotrophic growth (Bar-Even et al., 2012). For this, pyruvate was interconverted from acetyl-CoA by pyruvate:ferredoxin oxidoreductase (Charon et al., 1999). Although highly important, pyruvate:ferredoxin oxidoreductase gene was not classified as a core gene. In the cases of *Ca. hydrogenoformans* Z-2901 and *T. phaeum* DSM 12270, the pyruvate:ferredoxin oxidoreductase gene was not identified in the genomes. For the alternate reaction, formate C-acetyltransferase gene (pyruvate formate lyase, tph_c09600 and CHY_0877) present in the genome can be utilized for converting one acetyl-CoA with one formate to one pyruvate (Oehler et al., 2012).

To supply carbon skeletons, pyruvate reacts through reductive or oxidative branches of the incomplete tricarboxylic acid cycle, similar to most anaerobic bacteria. Specifically, the reductive branch was highly conserved throughout the acetogens (**Figure 5**). Initially, oxaloacetate, which is derived from pyruvate, was converted to fumarate via the reductive branch. Following this reaction, fumarate reductase, which was conserved in eight strains, synthesizes succinate from fumarate. However, all genes encoding the oxidative branch were classified as dispensable genes. The citrate synthase gene was located in only seven strains (**Figure 5**; Supplementary Table S5), while other enzymes, such as isocitrate dehydrogenase and 2-oxoglutarate synthase, were conserved, except in *Tr. primitia, Th. kivui, C. ljungdahlii*, and *C. autoethanogenum*. Among the acetogens, the least conserved enzyme associated with the tricarboxylic acid cycle was succinyl-CoA synthetase. In all acetogens, succinyl-CoA synthetases were located with the incomplete tricarboxylic acid cycles, which were composes of formations, with one direction leading to the formation of 2-oxoglutarate or succinyl-CoA from citrate and the other direction leading to the formation of fumarate or succinate from acetyl-CoA.

Central metabolic pathways, such as the glycolysis pathway, the pentose phosphate pathway, and the shikimate biosynthetic pathway, were highly conserved in all acetogens for nucleotide and amino acid biosynthesis (**Figure 5**). To produce the pentose phosphate for RNA and DNA precursors, the pentose phosphate pathway and gluconeogenesis must be utilized with related core genes. The shikimate pathway was also used in early steps for biosynthetic production of cofactors (folate), electron-transfer components (quinones), and aromatic amino acids (phenylalanine, trypsin, and tryptophan). All parts of these pathways were conserved, except for *aroD* genes, which were absent in the *Tr. primitia* genome (**Figure 5**; Supplementary Table S5). For the production of valine, leucine, and isoleucine from acetyl-CoA, acetolactate synthase, ketol-acid reductoisomerase (IlvC), and dihydroxy-acid dehydratase (IlvD) are required, which were conserved in all 14 acetogens (**Figure 5**). Following acetyl-CoA conversion, these conserved enzymes convert pyruvate into branched-chain amino acids.

## Cofactor Biosynthetic Pathways

Several enzyme-cofactor interactions are heavily involved in the Wood-Ljungdahl pathway, including THF, corrinoid iron-sulfur protein, and molybdopterin cofactor, which play key roles in one-carbon transfer for synthesizing acetyl-CoA from CO_2_/H_2_ (Drake, 1994; Ragsdale, 2008; Ragsdale and Pierce, 2008). Under the circumstances, genes encoding enzymes involved in the biosynthesis of cofactors should be present in the genome for pure cultures of CO/CO_2_-dependent chemolithotrophs without supplementation of the required cofactors.

**FIGURE 6:**
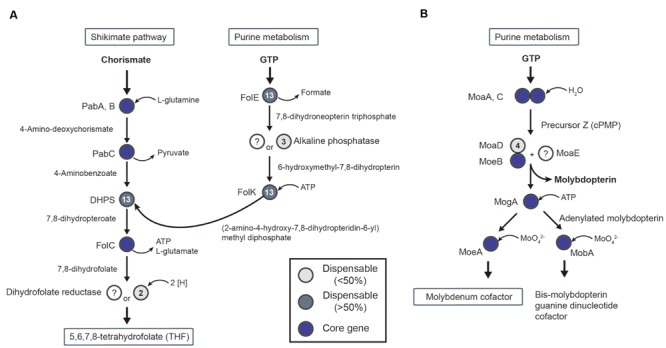
**Conserved pathway of cofactor biosynthesis in acetogens.** Pathways for tetrahydrofolate **(A)** and molybdenum cofactor **(B)** biosynthesis are shown with genes that are represented as core genes (blue circles), lesser conserved dispensable gene (<50%, light gray circles), and highly conserved dispensable genes (>50%, dark gray circles).

First, THF is important for the transformation of methyl-tetrahydrofolate following reduction of CO_2_. For THF synthesis, the *de novo* synthesis pathway begins with chorismate and guanosine triphosphate from the shikimate pathway and purine metabolism, respectively. All required genes were present in the core-gene set, except for two genes (**Figure 6A**): dihydrofolate reductase (DHR) and alkaline phosphate. Specifically, DHR was missing in most of the acetogens. A possible alternative enzyme is an oxygen-insensitive nitroreductase (Tph_c13060) for DHR (Oehler et al., 2012). The nitroreductase genes are core genes in acetogens, and studies of oxygen-insensitive nitroreductase reported evidence of DHR activity (Vasudevan et al., 1992).

In the steps of formate synthesis, selenocysteine FDH requires the molybdopterin cofactor to catalyze the reduction of CO_2_ to formate (Ragsdale and Pierce, 2008). The biosynthetic pathway associated with the molybdopterin cofactor is shown in **Figure 6B**. The first steps, catalyzed by MoaA and MoaC, use guanosine triphosphate to synthesize the precursor Z, followed by molybdopterin synthesis by MoaD, MoeB, and MoaE (**Figure 6B**). Interestingly, the gene encoding MoaE was not reported in any acetogens, including *M. thermoacetica* (Pierce et al., 2008). A predicted alternative enzyme is cysteine desulfurase (EC 2.8.1.7), which was located in all 14 acetogen genomes and uses a sulfur donor, such as MoaD, for molybdopterin synthesis (Mihara et al., 2002).

Cobalamin is a central cofactor in the Wood-Ljungdahl pathway, given that acetyl-CoA synthase reactions are cobalamin dependent. Although pathways for cobalamin biosynthesis were reported in *M. thermoacetica* (Pierce et al., 2008), the pathway has not been fully elucidated. The genes encoding cobalamin biosynthesis are located as a large gene cluster in the genome (Köpke et al., 2010; Oehler et al., 2012; Poehlein et al., 2012). Two distinct cobalamin-biosynthesis pathways were reported as an anaerobic and an aerobic pathway (Rodionov et al., 2003). Comparative genome analysis indicated that the aerobic pathway was absent in all acetogen genomes; however, the *cobJ, cobM, cobH*, and *cobB* genes were highly conserved. Nevertheless, the anaerobic cobalt-insertion pathway was conserved in six strains (*A. woodii, E. limosum, C. autoethanogenum, C. ljungdahlii, C. scatologenes*, and *Th. kivui*). Previously, the ability to the produce vitamin B_12_ under autotrophic or methylotrophic conditions was evaluated in two strains (Stupperich et al., 1988; Lebloas et al., 1994). However, sirohydrochlorin cobaltochelatase (*cbiK*) and precorrin-3 synthase (*cbiL*) genes were missing in two strains (*C. aceticum* and *C. difficile*). In the case of the others, two more genes were missing from the anaerobic cobalt-insertion pathway (Oehler et al., 2012). Such genes only found in individual strains may exist due to the dependency on vitamin B_12_ during autotrophic growth.

## Perspectives and Conclusion

Acetogens inhabit diverse environments, temperatures, and pH conditions (Drake et al., 2006). Correspondingly, the genomes of acetogens comprise highly diverse metabolic and energy conservation systems (Schuchmann and Müller, 2014; Poehlein et al., 2015b). For example, an F_0_F_1_-type ATP synthase, a conserved energy generating component, was conserved with seven subunits in 13 strains, except for *E. limosum* (Supplementary Table S5). However, ion specificity for gradient-driven phosphorylation is quite different between the strains due to the sequence motif present in the gamma subunit (Krah et al., 2010). Normally, the gamma subunit binds H^+^ at a site between the carboxyl oxygen of a carboxylate and a backbone carbonyl of another amino acid (Pogoryelov et al., 2009). For Na^+^, four amino acid residues are conserved: Gln32, Val63, Ser66, and Thr 67 (Murata et al., 2005). Although subunit α and β were well conserved with high similarity, the ion-binding subunit gamma was diverse, with relatively low similarity throughout the acetogens, possibly due to the variations in environmental conditions.

Despite this genetic diversity, the Wood-Ljungdahl pathway, a central metabolic pathway, and cofactor-biosynthetic pathways are highly conserved to promote autotrophic growth. Together, these data and previously reported results (Becerra et al., 2014) suggested that the ability to perform acetogenesis was obtained by genetic transfer of core genes associated with the Wood-Ljungdahl pathway and remains interconnected with its own inherent metabolic and energy conservation systems. Similarly, gene-set enrichment analysis revealed that acetogens do not share special gene sets, with the exception of the Wood-Ljungdahl pathway and *fwdE*.

Additionally, we predicted missing enzymes and suggested possible alternative enzymes based on the information from each genome. This information can aid in understanding the basic model of acetogens. Although we predicted the conserved pathways associated with individual strains, several key pathways remain unclear and require biochemical confirmation. Furthermore, the mechanisms involved in chemolithoautotrophic growth, systematic energy conservation, and precisely regulating carbon and energy flux also remain unknown. Also, the reconstruction of genome-scale models will be also required for the prediction of phenotypes and biosynthesis of value-added products of interest from syngas. In order for this to happen, the small differences found in conserved and alternative biochemical pathways can be used to optimize the genetic network to efficiently utilize the optimal enzymes or to convert optimal non-acetogenic microorganisms into novel acetogens.

## Author Contributions

JS and B-KC conceived and designed the analyses. JS, YS, and YJ performed the analyses. JS and B-KC wrote the paper. All authors approved the final manuscript.

## Conflict of Interest Statement

The authors declare that the research was conducted in the absence of any commercial or financial relationships that could be construed as a potential conflict of interest.

## Footnotes

1 ftp://ftp.ncbi.nih.gov/genomes/genbank/bacteria
