# Supplementary material for: Analysis of the Core Genome and Pan-Genome of Autotrophic Acetogenic Bacteria
Source: Front Microbiol. 2016 Sep 28;7:1531. doi: 10.3389/fmicb.2016.01531 (PMC5039349; doi:10.3389/fmicb.2016.01531)
Supplement: Supplementary file 7 [file Image_1.PDF]

1 Supplementary Figures and Tables

1.1 Supplementary Figures

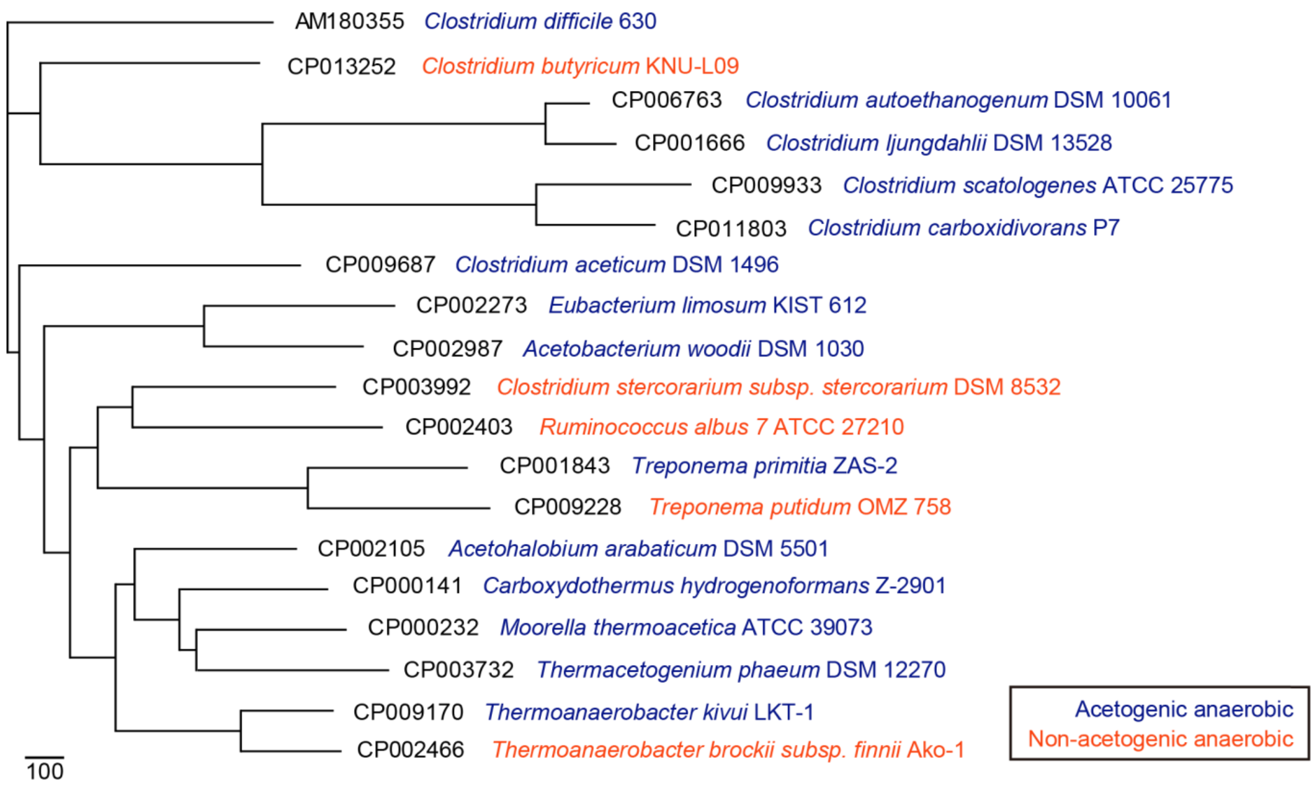

Supplementary Figure S1. Phylogenetic tree consisting of 14 acetogens and 5 non-acetogen (*Clostridium butylicum* KNU-L09, CP13252; *Clostridium stercorarium subsp. stercorarium* DSM 8532, CP003992; *Ruminococcus albus 7* ATCC 27210, CP002403; *Treponema putidum* OMZ 758, CP009228; *Thermoanaerobacter brockii subsp. finnii* Ako-1, CP002466)
