# Supplementary material for: Analysis of the Core Genome and Pan-Genome of Autotrophic Acetogenic Bacteria
Source: Front Microbiol. 2016 Sep 28;7:1531. doi: 10.3389/fmicb.2016.01531 (PMC5039349; doi:10.3389/fmicb.2016.01531)
Supplement: Supplementary file 8 [file Image_2.PDF]

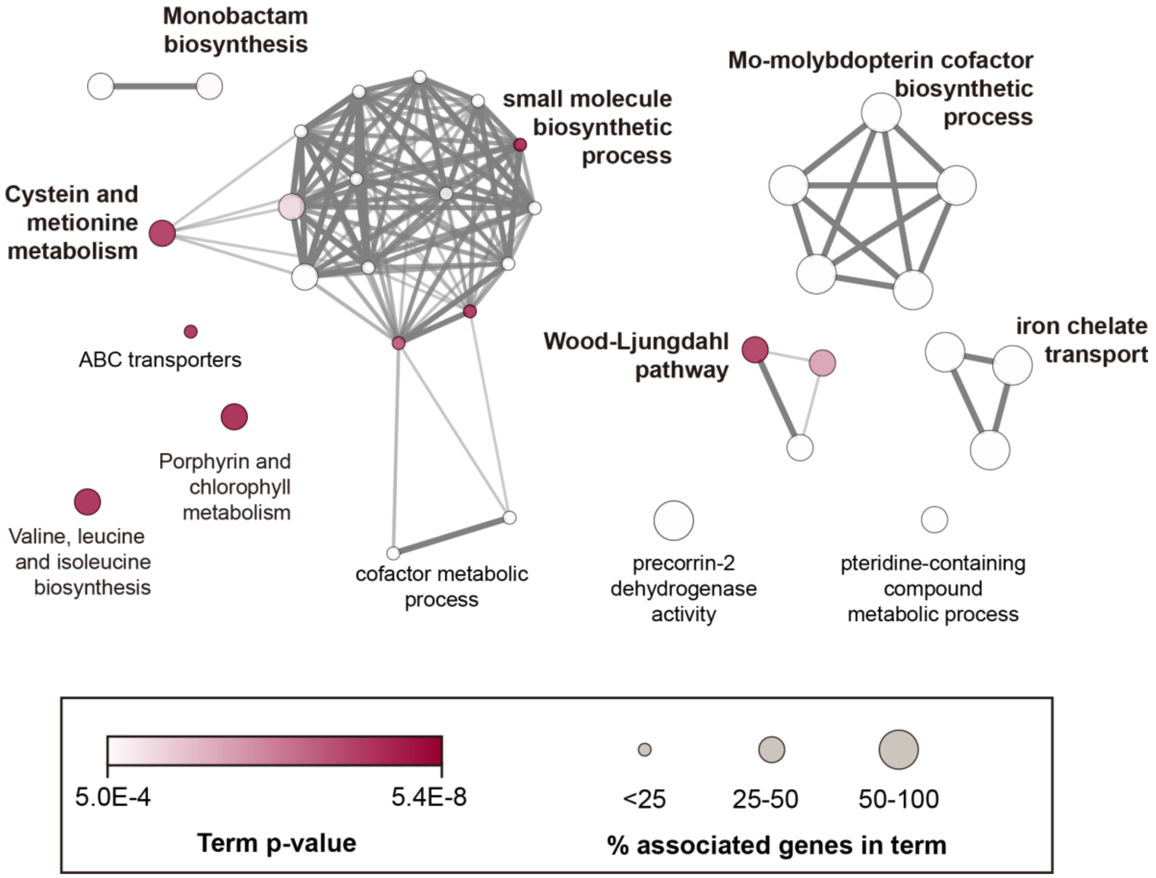

Supplementary Figure S2. Functionally grouped network for the trimmed core genomes by non-acetogenic core genome. KEGG and GO terms, including biological process, molecular function, and cellular component, were represented together as nodes, and node sizes represent the genes percentage association with each term. Significantly related terms were highly contacted, and functionally related nodes were partially overlapped. The most significant terms were only annotated in groups. A Bonferroni corrected $p < 0.05$ was considered the cut-off criterion. Term enrichment significance was represented by color.
